# Role of sarcomeric gene polymorphisms on left ventricular dysfunction in coronary artery disease patients

**DOI:** 10.1186/1755-8166-7-S1-P112

**Published:** 2014-01-21

**Authors:** Surendra Kumar, Avshesh Mishra, Anshika Srivastava, Naveen Garg, Surendra Kumar Agarwal, Shantanu Pande, Balraj Mittal

**Affiliations:** 1 Department of Genetics, Sanjay Gandhi Post Graduate Institute of Medical Sciences (SGPGIMS), Lucknow-UP, India; 2 Department of Cardiology, Sanjay Gandhi Post Graduate Institute of Medical Sciences (SGPGIMS), Lucknow-UP, India; 3 Department of CVTS, Sanjay Gandhi Post Graduate Institute of Medical Sciences (SGPGIMS), Lucknow-UP, India

## Background

Coronary artery disease (CAD) is a major cardiac disease in humans. Many CAD patients develop left ventricle dysfunction (LVD), leading to congestive heart failure. Mutations in several genes including those encoding sarcomeric proteins such as *MYBPC3*, *TNNT2*, *and TTN* are common genetic cause of hereditary cardiac myopathies. An intronic 25-bp deletion in *MYBPC3* at 3’ region is associated with dilated (DCM) and hypertrophic (HCM) cardiomyopathies in Southeast Asia. We sought to determine the role of *MYBPC3* 25bp, *TNNT2* 5bp and *TNN* 18bp ins/del polymorphisms on LVD in CAD patients.

## Methods and results

The study included 200 healthy controls and 988 consecutive patients with angiographically confirmed CAD. Among them, 253 with reduced ejection fraction (LVEF <45%) were categorized as having LVD. *MYBPC3* 25bp, *TNNT2* 5bp and *TNN* 18bp ins/del polymorphisms were determined by polymerase chain reaction. Our results showed that *MYBPC3* 25bp deletion was significantly associated with CAD as well as LVD (healthy controls v/s CAD; p value = **0.003**; OR=**4.08**, healthy controls v/s LVD; p value <**0.0001**; OR=**6.67** and Non-LVD v/s LVD; p value = **0.031**; OR=**1.67**). The *TNNT2* 5bp and TNN 18bp polymorphisms were not found to be associated with CAD (Pvalue=0.580, OR=0.88; Pvalue=0.795, OR=0.91; respectively) or LVD (Pvalue=0.146, OR=1.35; Pvalue=0.935, OR=0.97 respectively) when compared to controls.

## Conclusions

The frequency of *MYBPC3* DW genotype and D allele was associated with LVD implying that genetic variants of *MYBPC3* encoding mutant structural sarcomeric protein could increase susceptibility to left ventricular dysfunction. Therefore, 25bp deletion in *MYBPC3* may represent a genetic marker for cardiac failure in CAD patients.

